# Mutational Spectrum Drives the Rise of Mutator Bacteria

**DOI:** 10.1371/journal.pgen.1003167

**Published:** 2013-01-10

**Authors:** Alejandro Couce, Javier R. Guelfo, Jesús Blázquez

**Affiliations:** Departamento de Biotecnología Microbiana, Centro Nacional de Biotecnología (CNB-CSIC), Madrid, Spain; Université Paris Descartes, INSERM U1001, France

## Abstract

Understanding how mutator strains emerge in bacterial populations is relevant both to evolutionary theory and to reduce the threat they pose in clinical settings. The rise of mutator alleles is understood as a result of their hitchhiking with linked beneficial mutations, although the factors that govern this process remain unclear. A prominent but underappreciated fact is that each mutator allele increases only a specific spectrum of mutational changes. This spectrum has been speculated to alter the distribution of fitness effects of beneficial mutations, potentially affecting hitchhiking. To study this possibility, we analyzed the fitness distribution of beneficial mutations generated from different mutator and wild-type *Escherichia coli* strains. Using antibiotic resistance as a model system, we show that mutational spectra can alter these distributions substantially, ultimately determining the competitive ability of each strain across environments. Computer simulation showed that the effect of mutational spectrum on hitchhiking dynamics follows a non-linear function, implying that even slight spectrum-dependent fitness differences are sufficient to alter mutator success frequency by several orders of magnitude. These results indicate an unanticipated central role for the mutational spectrum in the evolution of bacterial mutation rates. At a practical level, this study indicates that knowledge of the molecular details of resistance determinants is crucial for minimizing mutator evolution during antibiotic therapy.

## Author Summary

Natural and laboratory populations of bacteria can readily give rise to strains with high mutation rates. The evolution of these mutator bacteria—of particular concern in clinical situations—has been understood exclusively in terms of their increased capacity to generate beneficial mutations, such as those that confer antibiotic resistance. Current models, however, have largely overlooked that each mutator allele increases only characteristic types of mutations, a prominent fact whose evolutionary consequences remain unexplored. Using laboratory *Escherichia coli* populations, we show that this mutational bias determines the competitiveness of different mutators across environments. Computer simulation showed that this effect can markedly influence the evolutionary fate of mutator alleles. These results indicate that this unrecognized factor can be a major determinant in the evolution of mutator bacteria and suggest future experimental approaches for improving antibiotic therapy design.

## Introduction

Despite their increased load of deleterious mutations [1], [2], mutator strains of bacteria are isolated routinely in laboratory and clinical settings [3]–[8]. Theory [9]–[11] and experiments [5], [12], [13] explain these observations as a consequence of genetic hitchhiking, whereby mutator alleles reach high frequency by being co-selected with linked beneficial mutations. Mutator evolution is therefore dependent on the absence of horizontal gene transfer [14] and the availability of adaptive mutations with substantial fitness effects [11] – conditions frequently met during adaptation to a host or during antibiotic therapy, which have been invoked to explain the prevalence of mutators among pathogenic bacteria [15]–[17].

Whereas genetic hitchhiking provides a satisfactory mechanism to explain how mutator bacteria can be selected, the precise mechanistic details of this process are still a matter of research. Some authors emphasize that mutator fixation involves many consecutive hitchhiking events, prompted by frequent environmental shifts [9], [18], [19] or by the concurrence of multiple beneficial mutations [10], [11]. Other authors, in contrast, consider mutator success as primarily the result of a single step in which one hitchhiking event takes mutator frequency from rareness to fixation [20]. Despite this theoretical effort, two major observations remain to be accounted for. First, although hitchhiking probability is predicted to increase with the extent of adaptive opportunity offered by the environment (i.e., the number and effects of available beneficial mutations) [14], [20], , selection experiments with comparable adaptive opportunity report contrasting frequencies for mutator emergence [4], [12], [22], [23]. Second, mutators of different strength are predicted to emerge according to the degree of adaptive opportunity [14], [21]; nonetheless, both clinical and laboratory observations show a marked bias towards strong mutators, particularly those caused by defects in the mismatch repair system (MMR) [17], [24]. To explain these deviations, it is argued that mutator alleles of other mutator strengths might be under-represented due to putative fitness disadvantages, and that MMR mutator mutants might be over-represented because they can also be selected for their high recombination rates [24].

An additional, previously unrecognized factor is that each mutator exhibits its own mutational spectrum, that is, mutation rate increase is limited to characteristic types of mutations [25]. This bias stems from the type of mutation avoidance mechanism that is altered in each mutator genotype; depending on which mechanism is affected, its failure will lead to a preferential increase in specific types of transitions, transversions or frameshifts [25]. In line with previous suggestions [12], [26], we hypothesized that if mutators and wild-type strains have differential access to specific beneficial mutations, they might produce mutants with different fitness levels, which could influence their evolutionary dynamics. Mutator alleles that on average generate stronger beneficial mutations will have a better chance to achieve fixation; conversely, those that more often produce weaker adaptive mutations will have limited spread. The fixation probability of a mutator allele would thus depend not only on its mutation rate, but also on its mutational spectrum.

Here we test these predictions by characterizing beneficial mutations from wild-type and knockout strains of the *mutT* (*ΔmutT*) and *mutY* (*ΔmutY*) antimutator genes of *Escherichia coli*. *mutT*-defective strains show a strong mutator phenotype, leading specifically to A·T→C·G transversions [25], whereas *mutY* defects lead to a moderate mutator phenotype that increases G·C→T·A transversions [25]. Both mutators were reported to arise spontaneously in evolution experiments with *E. coli* [4], [12]. Since beneficial mutations are exceedingly rare and difficult to detect, our experimental design focused on mutations that confer antibiotic resistance, which are particularly suitable for this kind of study. We show that according to their mutational spectra, wild-type and mutator strains can generate distinct fitness distributions for antibiotic-resistant mutants. Notably, these dissimilarities can significantly alter the competitive abilities of mutators against the wild-type strain, as measured in direct competition experiments. Furthermore, using computer simulation of a simple population genetics model, we show that the hitchhiking dynamics is highly sensitive to average fitness deviations of the beneficial mutations with which mutator alleles hitchhike.

## Results/Discussion

### Colony size polymorphism of antibiotic-resistant mutants

**Figure 1:**
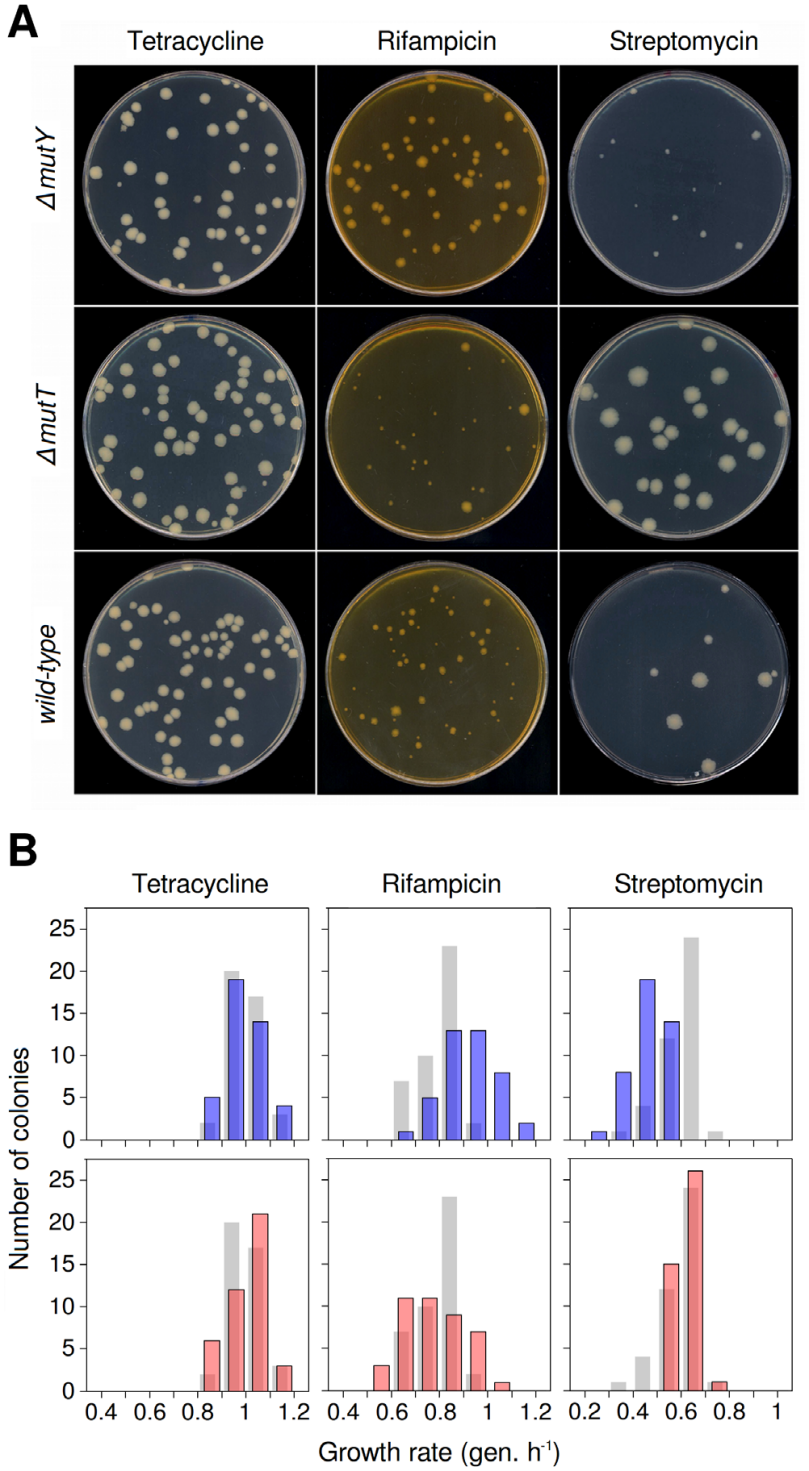
Mutational spectrum effects on generation of antibiotic-resistant mutants of wild-type and mutator *E. coli.* (A) Characteristic colony size polymorphism generated by each strain in tetracycline (left), rifampicin (centre) and streptomycin (right). Resistance mutations to rifampicin and streptomycin impair bacterial growth to varying degrees, and are produced differentially according to mutational spectra. Tetracycline resistance serves here as a control, as resistance mutations in these strains show little variability for fitness. (B) Fitness distributions for antibiotic-resistant mutants generated by each strain. Histograms represent 42 independent growth rate estimations. Blue bars, *ΔmutY* (top); pink bars, *ΔmutT* (bottom). Wild-type reference values (grey bars) are placed behind those of each mutator. The greater diversity observed here compared to Figure 1A is a result of the larger number of independent cultures.

To test whether mutational spectrum differences translate into significant fitness differences, beneficial mutations were selected by plating cultures of wild-type, *ΔmutT* and *ΔmutY* strains in three antibiotics: rifampicin, streptomycin and tetracycline. Rifampicin- and streptomycin-resistant mutants showed marked colony size polymorphism (Figure 1A). Resistance to these antibiotics arises readily through alterations in their targets, the RNA polymerase [27] and the 30S ribosomal subunit [28], respectively. Since many alterations can affect the function of these essential machineries to different degrees, it is unsurprising that resistance mutants show growth differences. Consistent with the predictions of our hypothesis, wild-type and mutator strains displayed distinct colony size polymorphism in each antibiotic (Figure 1A). To test whether access to beneficial mutations with varying selection coefficients was the only factor responsible for differences among strains, we used strains with an insertion bearing a tetracycline-resistance gene and its constitutive repressor [29]. As the probability of achieving resistance to high tetracycline concentrations through point mutation in *E. coli* is negligible (mutant frequency <10^−9^), resistance arises here only through loss-of-function mutations, which relieve the repression. All of these loss-of-function mutations can be considered equivalents, and no fitness differences are thus anticipated among tetracycline-resistant mutants. Indeed, these mutants showed little growth polymorphism (Figure 1A). Small differences, apparently independent of the mutator background, probably reflect phenotypic lag or other sources of phenotypic variability.

### Distribution of fitness effects on plate of antibiotic-resistant mutants

To quantify the fitness distribution of the mutants generated by each mutator, we randomly selected 42 independent resistant colonies from each combination of genotype and antibiotic, and measured growth rate as a proxy for Darwinian fitness. It is important to remark that we are dealing with the fitness distribution after selection, not the intrinsic fitness distribution; and so it should be understood in what follows. In rifampicin, the distribution of mutants generated by the *ΔmutY* strain is shifted toward higher growth rates compared with both those of wild-type and *ΔmutT* strains (Figure 1B, centre), whereas in streptomycin the situation is the reverse (Figure 1B, right) (*n* = 42, P<0.0001, Kolmogorov-Smirnov's one-sided two-sample test, in all cases). These results suggested that G·C→T·A substitutions in the *rpoB* gene (the characteristic transversion increased in this mutator) produced alleles encoding a high-fitness rifampicin-resistant RNA polymerase; coincidentally, the same transversion generated *rpsL* alleles encoding a low-fitness streptomycin-resistant ribosomal protein S12. Similar reasoning could be applied to the *ΔmutT* strain results, which preferentially raises the A·T→C·G transversion. In the case of tetracycline, no significant differences were found between wild-type and any mutator strains (Figure 1B, left) (*n* = 42, P>0.18, Kolmogorov-Smirnov's two-sided two-sample test).

### Characterization of the genetic basis of antibiotic resistance

**Figure 2:**
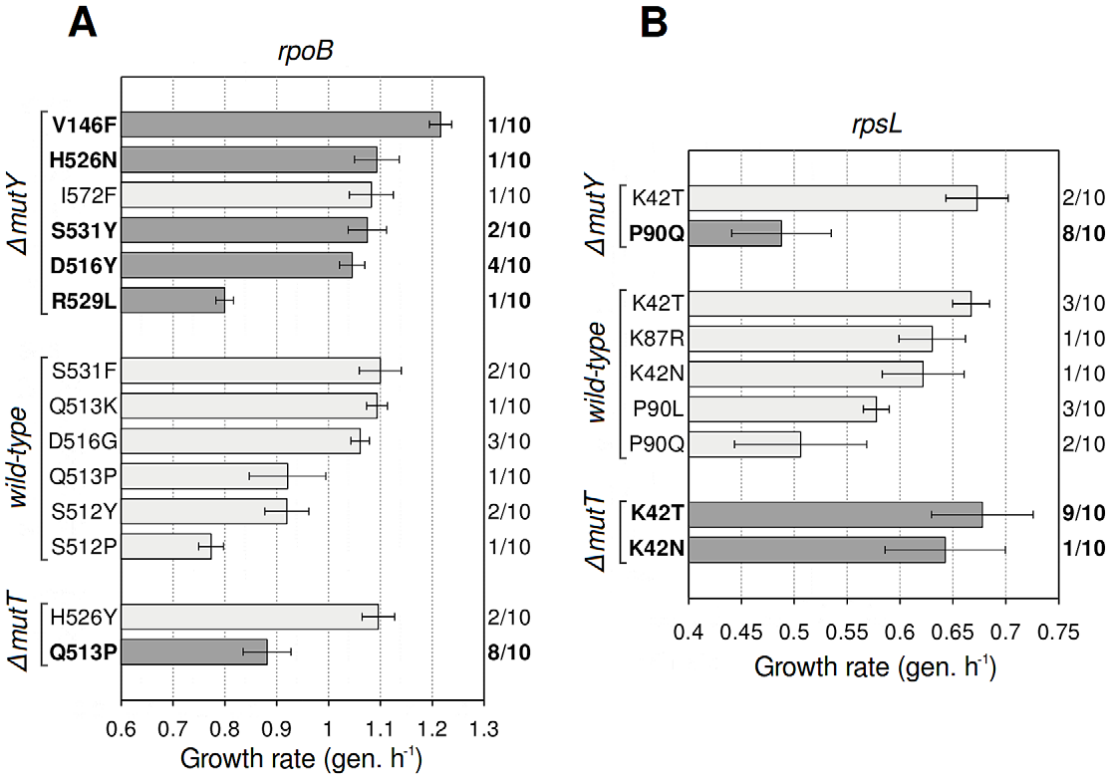
Antibiotic resistance mutations generated by wild-type and mutator strains. Growth rate (mean ± SD; *n* = 3) of mutants bearing the specified mutation. Dark grey bars indicate mutations corresponding to the mutational spectrum of each mutator. Frequency of occurrence of each mutation is shown (right axis). All substitutions observed are described to confer antibiotic resistance. (A) Mutations in *rpoB*, which confer rifampicin resistance. (B) Mutations in *rpsL*, which confer streptomycin resistance.

To confirm our interpretation, we randomly picked 10 colonies from each combination of antibiotic and strain, sequenced their *rpoB* and *rpsL* genes, and measured growth rates (Figure 2). Several G·C→T·A substitutions found in *rpoB* can explain the higher fitness of rifampicin-resistant *ΔmutY* mutants, supporting our hypothesis. The highest-fitness class (Figure 1B, centre) is likely to be composed of V146F mutants, the fastest-growing mutant detected (Figure 2A); other high-fitness mutations that resulted from this transversion were H526N and S531Y. The fitness distribution in rifampicin-resistant *ΔmutT* mutants can be explained, at least in part, by an A·T→C·G substitution that produces the low-fitness mutation Q513P. Among streptomycin-resistant mutants, idiosyncratic transversions in *rpsL* similarly help to explain the fitness differences. The low fitness of streptomycin-resistant *ΔmutY* mutants probably reflects predominance of the P90Q mutation (8/10 mutants tested), whereas the high fitness of *ΔmutT* mutants might be due to prevalence of the K42T mutation (9/10). As a brief remark, 3/10 of the streptomycin-resistant mutants in the wild-type background carry the substitution P90L. This mutation is known to prevent growth in the absence of the antibiotic [28], and offers a suggestive example of how spectra can determine the access to mutations with differences not only in fitness, but also in other related properties.

The biochemical bases of the fitness cost of both rifampicin and streptomycin resistance have been discussed elsewhere. The costs of *rpoB* mutations are explained by the impairment of the transcription activity of the RNA polymerase [30], [31]. Similarly, it has been long established that *rpsL* streptomycin resistance mutants exhibit hyperaccurate translation, resulting in a slower rate of protein synthesis and consequently, in a slower growth rate [32].

### Average fitness of antibiotic-resistant mutator genotypes

**Figure 3:**
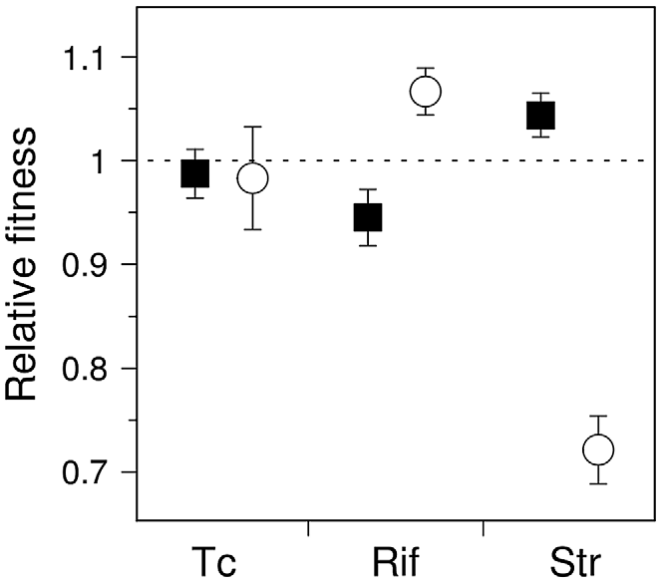
Competition experiments for antibiotic-resistant mutants of wild-type and mutator *E. coli*. Fitness (mean ± SD; *n* = 4) of antibiotic-resistant mutants generated by *ΔmutY* (circles) and *ΔmutT* (squares). Fitness was calculated relative to the wild-type strain; dashed line indicates equal competitive ability. Note that the relative abundance of competing mutants is anticipated to vary among replications, as mutants were generated stochastically during the growth period prior to competition.

It is tempting to assume that if the fitness distribution of a given mutator is altered compared to that of wild-type, the average fitness of that mutator will change accordingly. These distributions nonetheless have distinct shapes and degrees of overlap (Figure 1B); it is thus of interest to determine the extent to which these differences translate into an overall fitness difference between each mutator and the wild-type genotypes. Direct competition experiments between mutators and wild-type strains in rifampicin and streptomycin showed significant differences in mean fitness (Figure 3) (*n* = 4, P<0.012, Mann-Whitney U-test, one-sided, all cases), confirming the predictions made by visual examination of Figure 1A.

### Mutational spectrum effect on hitchhiking dynamics

Our data provide evidence that in a specific environment, distinct mutators generate their own fitness distribution among newly-arising mutants, which can influence their competitive ability. Theory predicts the fixation probability of a mutator allele to be dependent on the selection coefficient of the driver allele with which the mutator hitchhikes [11], [20]. According to our results, this coefficient can vary substantially depending on the mutational spectrum; the mutational spectrum should thus have some effect on the hitchhiking dynamics. To estimate how large this effect could be, we used simple computer simulations based on previous studies [10], [11], [20]. Briefly, we simulated the basic scenario of a non-mutator bacteria population growing in batch culture, where only one beneficial mutation is needed to achieve full adaptation. Mutators are generated at a constant rate, and produce the adaptive mutation with a 100-fold higher probability than the wild-type strain. Once the mutation is fixed in either background, the simulation ends. The mutational spectrum effect (*σ*) was introduced as a multiplicative factor to modify the selection coefficient (*s*) of the driver allele only on the mutator background (see Material and Methods).

**Figure 4:**
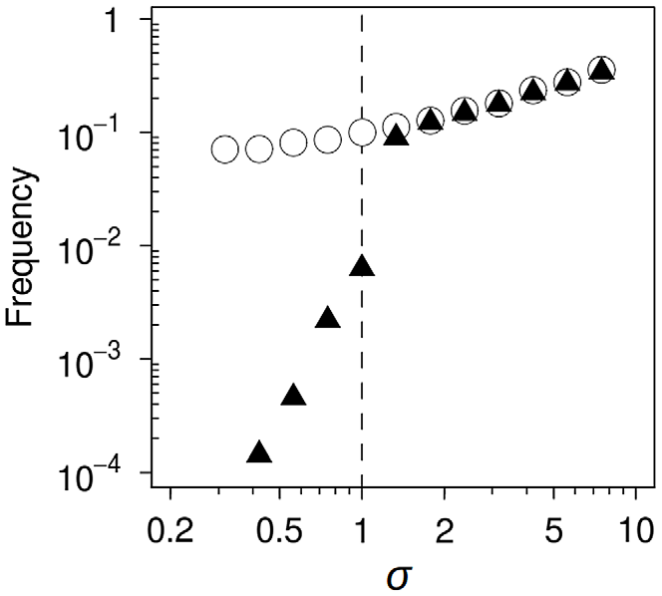
Computer simulation of mutational spectrum effects on hitchhiking dynamics. The mutational spectrum can bias the average fitness of mutants produced by a mutator compared to those produced by the wild-type strain. We modelled this bias as a multiplicative factor (*σ*), such that when *σ* = 1, there is no difference between the selective advantage conferred by the beneficial mutation on either background. Circles represent the frequency of trials in which a mutator allele escaped drift; triangles show the frequency of trials in which this allele reached fixation. Only slight variations in *σ* led to a profound effect on hitchhiking dynamics (see text for a definition of *σ* and a detailed explanation of the dynamics).

**Figure 5:**
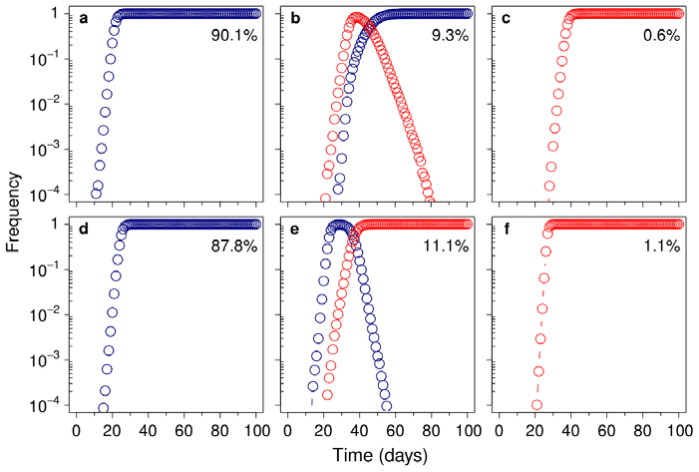
Mutational spectrum effect on hitchhiking dynamics. Representative runs showing the change in frequency of the adapted wild-type (*100*) (blue) and the adapted mutator (*101*) (red), for *σ* = 1 (A–C) and *σ* = 1.8 (D–F). The beneficial mutation confers a 10% increase in relative fitness (*s_b_* = 0.2), whereas each deleterious mutation reduces it by 2% (*s_d_* = 0.04). All the results can be classified into three main cases: only the adapted wild-type escapes drift (A and D), both genotypes escape drift (B and E), or only the adapted mutator escapes drift (C and F). Numbers are the percentage of runs (of 35,000) that correspond to each case. A–C, Without mutational spectrum effects (*σ* = 1), in 90.1% of cases the much larger wild-type subpopulation generates an adapted genotype that reaches fixation before any adapted mutator is established (A). In the cases in which both adapted genotypes coexist (9.3% of the total), the wild-type is always fixed, due its lower deleterious load (B). This confines mutator success to the remaining 0.6% of cases, in which they are able to reach fixation before any adapted wild-type genotype escapes drift (C). D–F, When mutational spectrum effects are taken into account (*σ* = 1.8), mutators not only escape drift more frequently but, due to their higher fitness, they are able to reach fixation even in the presence of an adapted wild-type (E). As a result, there is a marked increase in the percentage of cases in which mutators become fixed (11.1%+1.1% = 12.2% versus 0.6% when *σ* = 1).

The simulations showed that *σ* exerts a modest influence on the establishment of mutator genotypes bearing the adaptive mutation (i.e., on the probability that they escape random drift) (Figure 4). This effect is not surprising, as the probability of a beneficial mutation surviving drift is approximately *2s* [33]. In contrast, *σ* had a notable effect on the fate of established genotypes en route to fixation. In the absence of mutational spectrum effects (*σ* = 1), a mutator genotype bearing the adaptive mutation can only succeed if it reaches fixation before any adapted wild-type bacteria escapes drift; it will otherwise always be outcompeted due to its increased deleterious mutation load (Figure 5, upper row). When the average *s* of the driver allele is lower on the mutator background (*σ*<1), there are no qualitative changes. Success is further hindered because drift is more intense and time to fixation is longer, extending the period available for the establishment and subsequent selective sweep of an adapted wild-type strain. In contrast, when *σ*>1 a threshold appears above which the population dynamics switches. This threshold is determined by the value of *σ* that offsets the increased deleterious load of mutator genotypes. Above this value, the adapted mutator is fitter than its wild-type counterpart, and therefore needs only to escape drift to reach fixation (Figure 5, lower row); as a consequence, fixation probability rises sharply (Figure 4). It is worth noting that, since the deleterious load is as small as the order of magnitude of the mutation rate [33], only a slight spectrum-dependent fitness advantage is needed to substantially increase mutator success.

Remarkably, the non-linear response of fixation probability to *σ* implies that previous models could have been underestimating the likelihood of mutator success by several orders of magnitude. This is clearly illustrated in Figure 4 where a change from *σ = 0.56* to *σ = 1.33,* which is equivalent to a change in relative fitness [37] from *w = 0.96* to *w = 1.03,* represents a ∼196-fold increase in fixation probability.

### Conclusions

Selection of mutators has been understood exclusively in terms of the increased number of beneficial mutations they generate [5], [10]. Here we show that not only the number but also the type of these linked mutations are relevant. Our results indicate that mutator alleles can bias the average selection coefficient of the beneficial alleles with which they hitchhike. Besides, they suggest that the magnitude of this effect can easily be sufficient to drastically modify their probabilities to reach fixation. The only requirement for this bias is that the locus or loci under selection produce mutants with some variability for fitness. This is a fairly permissive condition, likely to be satisfied in several adaptive scenarios. Examples in which resistant mutants with varying degrees of fitness are commonly found include bacteriophage [34] and antibiotic resistance [35], considered major drivers of mutator evolution [8], [15]. It will be of interest to determine whether the observed relative abundance of each mutator is explained, at least in part, by the effect of its mutational spectrum on successive mutations undergone during adaptation. In the context of clinical infections, future work should address the extent to which antibiotic therapies show different propensities to select for mutator bacteria. Since mutators are recognized as a risk factor for treatment failure [15]–[17], this knowledge could help to improve the design of safer therapeutic strategies.

The fact that even slight mutational spectrum effects markedly alter hitchhiking, together with the apparent commonness of circumstances that potentially allow this to happen, lead us to conclude that the mutational spectrum is a major factor in the evolution of mutators in laboratory and clinical populations of bacteria [3]–[8], as well as in certain cancers [36].

## Materials and Methods

### Strains and culture conditions

Strains are *E. coli* MG1655 derivatives, obtained from Dr. I. Matic [29]. Bacteria were grown on Luria broth (LB) or LB agar plates (37°C). Antibiotics used were tetracycline (15 mg/L), rifampicin (100 mg/L) and streptomycin (100 mg/L). Incubation time was 24 h, except for streptomycin plates (42 h).

### Growth rate assays

Overnight cultures of each strain were plated on each antibiotic at appropriate dilutions to ensure low colony density (∼50/plate). After incubation, independent colonies were picked at random (by proximity to an arbitrary point) and resuspended in saline solution. Population size (*N*) was estimated from viable counts by subsequent dilution and plating. Assuming that each colony originated from a single cell, generation number was calculated as *log_2_N* and the growth rate expressed as number of generations per hour. Note that this measurement integrates growth rate over all growth phases.

### Competition experiments

Overnight cultures of each mutator and wild-type strain were mixed at a ratio based on their mutation rates, plated on antibiotic, and allowed to compete for 24 or 42 h. Initial competitor frequency was calculated by estimating the resistant-mutant frequency of each overnight culture. To distinguish them from the wild-type bacteria, mutators carried antibiotic resistance markers [29], which entailed no significant fitness cost in these conditions (*n* = 4, P>0.28, Mann-Whitney U-test, two-sided, both cases). After incubation, agar plates were washed in saline solution and the final mutator∶wild-type ratio obtained by plating on LB agar and selective media. Relative fitness was estimated using a standard formula [37].

### Computer simulation

#### (i) General description of the algorithm

We simulated the serial passage of an asexual population, originated from a single wild-type cell, in a laboratory environment in which only one beneficial mutation is available. The overall scheme is the same as in Tenaillon *et al.* [11], and can be summarized in two phases: (1) population growth, which includes differential reproduction and mutation during a number of generations, and (2) a random sampling process. Programming was carried out using the R programming language [38] (source code available from www.free-bit.org/public/acouce/).

Genotypes are defined by three loci: *B*, *D* and *M.* The first locus codes for the absence (*b = 0*) or presence (*b = 1*) of the beneficial mutation, the second locus controls the number of accumulated deleterious mutations (*d*), and the last encodes the absence (*m = 0*) or presence (*m = 1*) of a mutator allele with a 100-fold increase in mutation rate. In this model, the wild-type genotype, for example, will be unequivocally denoted by *000*. In each generation, non-mutator individuals reproduce deterministically in accordance with the absolute fitness (*r_bd0_*) defined by their genotypes (*bd0*) as:
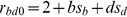
(1)where *s_b_* and *s_d_* are the beneficial and deleterious selection coefficients, respectively. The population grows exponentially until its size exceeds a value of 10^8^ individuals. Then, a random sample of 10^5^ individuals is taken, a bottleneck of moderate strength [39], after which growth is resumed. Simulations end when the beneficial mutation is completely fixed in the wild-type or in the mutator background.

#### (ii) Modeling the mutational spectrum effect

As we showed experimentally, the mutational spectrum can affect the average selection coefficient of the beneficial mutation with which a mutator allele ‘hitches a ride’. To implement this effect, we defined the the absolute fitness of mutators (*bd1*) as:
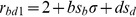
(2)where *σ* represents this mutational spectrum effect.

Care has been taken to ensure that equation (2) is only applied to adapted mutator genotypes (*1d1*) generated from a mutator background (*0d1*), but not to those generated in the rare but non-negligible case of an already adapted wild-type background (*1d0*) mutating the *M* locus.

#### (iii) Modeling transitions among genotypes

In each generation, after reproduction, all types of mutations are implemented by generating Poisson-distributed pseudorandom numbers [40], according to the population size of each genotype and a defined set of mutation rates (which act as the parameter of the Poisson distribution). Wild-type mutation rates were chosen to be in the range of estimated values for *E. coli,* 10^−7^ for beneficial mutations [41], 10^−4^ for deleterious mutations [42] and 5×10^−6^ for acquiring a mutator genotype [1]. Lethal mutation was introduced at a rate of 10^−5^, based on previous reports [10], [11]. Back-mutation was denied. One order of magnitude variation on these parameters showed no qualitative changes relative to the results shown here.
